# Does *Glycine max *leaves or *Garcinia Cambogia *promote weight-loss or lower plasma cholesterol in overweight individuals: a randomized control trial

**DOI:** 10.1186/1475-2891-10-94

**Published:** 2011-09-21

**Authors:** Ji-Eun Kim, Seon-Min Jeon, Ki Hun Park, Woo Song Lee, Tae-Sook Jeong, Robin A McGregor, Myung-Sook Choi

**Affiliations:** 1 Center for Food and Nutritional Genomics Research, Kyungpook National University, Daegu, Republic of Korea; 2 Department of Food Science and Nutrition, Kyungpook National University, Daegu, Republic of Korea; 3 Division of Applied Life Science (BK 21 Program), EB-NCRC, Institute of Agriculture and Life Science, Graduate School of Gyeongsang National University, Jinju, Republic of Korea; 4 Eco-Friendly Biomaterial Research Center, Korea Research Institute of Bioscience and Biotechnology, Jeongeup, Republic of Korea; 5 National Research Laboratory of Lipid Metabolism & Atherosclerosis, Korea Research Institute of Bioscience and Biotechnology, Daejeon, Republic of Korea

**Keywords:** atherosclerosis, cholesterol, clinical trial, hydroxyl citric acid, soybean leaves, weight-loss

## Abstract

**Background:**

Natural food supplements with high flavonoid content are often claimed to promote weight-loss and lower plasma cholesterol in animal studies, but human studies have been more equivocal. The aim of this study was firstly to determine the effectiveness of natural food supplements containing *Glycine max *leaves extract (EGML) or *Garcinia cambogia *extract (GCE) to promote weight-loss and lower plasma cholesterol. Secondly to examine whether these supplements have any beneficial effect on lipid, adipocytokine or antioxidant profiles.

**Methods:**

Eighty-six overweight subjects (Male:Female = 46:40, age: 20~50 yr, BMI > 23 < 29) were randomly assigned to three groups and administered tablets containing EGML (2 g/day), GCE (2 g/day) or placebo (starch, 2 g/day) for 10 weeks. At baseline and after 10 weeks, body composition, plasma cholesterol and diet were assessed. Blood analysis was also conducted to examine plasma lipoproteins, triglycerides, adipocytokines and antioxidants.

**Results:**

EGML and GCE supplementation failed to promote weight-loss or any clinically significant change in %body fat. The EGML group had lower total cholesterol after 10 weeks compared to the placebo group (p < 0.05). EGML and GCE had no effect on triglycerides, non-HDL-C, adipocytokines or antioxidants when compared to placebo supplementation. However, HDL-C was higher in the EGML group (p < 0.001) after 10 weeks compared to the placebo group.

**Conclusions:**

Ten weeks of EGML or GCE supplementation did not promote weight-loss or lower total cholesterol in overweight individuals consuming their habitual diet. Although, EGML did increase plasma HDL-C levels which is associated with a lower risk of atherosclerosis.

## Background

Obesity is a major threat to worldwide public health and is recognized as a major factor contributing to insulin resistance, type 2 diabetes, hyperlipidemia, hypertension, cardiovascular disease and all-cause mortality [1,2]. A modest weight loss in obese or overweight individuals is reported to be associated with decreased risk of co-morbidities and mortality [3]. Furthermore, reduction in total cholesterol or low-density lipoprotein cholesterol (LDL-C) through diet therapy or drug administration has been shown to decrease the risk of cardiovascular disease [4].

Natural food supplements are widely used by individuals for potential health benefits such as weight-loss [5,6] and lower cholesterol [7-10]. However, the evidence for the effectiveness of natural food supplements to promote weight-loss and improve health is largely derived from animal studies [5]. Therefore, it is essential randomized double-blind placebo-controlled trials **(**RCTs) are conducted to determine the effectiveness of natural food supplements to promote weight-loss and improve cholesterol levels [7].

The potential health-benefits of soy supplements have been extensively studied [7-9,11]. Soy supplementation has been reported to promote weight loss [11] and reduce plasma cholesterol [9]. Past studies have suggested soy may promote weight-loss via several mechanisms including inhibition of adipogenesis, appetite suppression, displacement of fat intake and increased satiety [11]. Soy is also suggested to have an effect on plasma cholesterol, via increased plasma high-density lipoprotein cholesterol (HDL-C) and soy may also protect low density lipoprotein (LDL) from oxidation. The main flavonoids in soy, daidzein and genistein closely mimic the chemical structure of estrogen, therefore soy may exert biological effects via binding to estrogen receptors [8]. Soy leaves contain different flavonoid and polyphenol profiles compared to soy beans, which some have suggested may provide unique additional health benefits compared to soybean based natural supplements [12,13]. Despite the intriguing evidence from *in-vitro *and animal studies on the mechanisms via which soy supplements may promote weight-loss [8] and improve plasma cholesterol [9], RCTs have been far more equivocal [7,11] and no RCTs have evaluated the effectiveness of a soy leaves based supplement to promote weight-loss or lower plasma cholesterol.

Natural food supplements containing *Garcinia cambogia *extract (GCE) have also been widely promoted as potential weight-loss aids and potential cholesterol lowering agents [5]. The effects of *Garcinia cambogia *have largely been attributed to it's rich (-)-hydroxycitric acid (HCA) content. A recent meta-analysis of nine trials suggested *Garcinia cambogia*/HCA supplementation may cause short-term weight-loss, but the clinical relevance still remains to established [14]. Several mechanisms have been suggested, including inhibition of energy metabolism and appetite suppression [15]. For example, HCA has been shown to increase the release of serotonin, a neurotransmitter implicated in the regulation of eating behaviour and appetite control [16]. Also HCA can act directly on adipocytes, causing lipid droplet dispersion and altering transcription [17]. Other bioactive components of *Garginia cambogia *including benzophenones are reported to reduce oxidative stress levels based on *in-vitro *experiments in human plasma, hence *Garginia cambogia *may protect against diseases associated with oxidative stress [18]. *Garginia cambogia *is also reported to suppress cholesterol and triglyceride accumulation in high fat diet fed mice [19]. However, evidence that *Garcinia cambogia can *improve blood lipid profiles or has antioxidant activity in humans is lacking [20].

RCTs of the effectiveness of HCA supplements to promote weight-loss have produced equivocal findings. The largest and most rigorous RCT to date found no significant difference in weight-loss between HCA and placebo supplementation [21]. Furthermore, a recent meta-analysis based on nine RCTs concluded HCA supplementation may promote only minimal short-term weight-loss, which appears to be of limited clinical relevance [14].

The aim of this study was to examine the effectiveness of soy (*Glycine max*) leaves, compared to *Garginia cambogia *or placebo supplementation on weight-loss and plasma cholesterol in overweight individuals consuming their habitual diet. We also examined whether either supplement had any beneficial effect on blood adipocytokines or antioxidant levels.

## Methods

### Subjects

Eighty six volunteers aged 20-60 years were recruited from the local community in Daegu (population 4.1 million), Republic of Korea. Inclusion criteria were overweight individuals with BMI > 23 and < 29. We used a lower BMI cut-off for overweight individuals compared to the World Health Organization overweight BMI criteria, because some reports suggest the prevalence of obesity related co-morbidities is higher in Asian cohort studies at a lower BMI cut-off [22]. Exclusion criteria included: pregnancy, smoking, serious illness, current treatment with any medications for the control of blood glucose levels, clinical or biochemical evidence of acute or chronic infection, hepatic dysfunction, chronic alcohol consumption, regular functional food supplement consumption which may affect the outcome of this study or any major surgery in the 6 months prior to the study. In addition, no subjects were actively using any other methods for weight reduction or control of blood lipids, including hypocaloric diet, anorexic drugs or lipid-lowering drugs. The study was performed in accordance with the Declaration of Helsinki. All subjects provided written informed consent prior to participating in this study. The study protocol was approved by the Kyungpook National University Human Research Committee (No. 2010-2).

### Study design

The study design was a randomized, double-blind, placebo-controlled trial to determine the effectiveness of extract of *Glycine max *leaves or *Garcinia cambogia *extract supplementation in overweight subjects to alter body composition, plasma cholesterol, lipids, adipocytokine or antioxidant levels. The primary outcome measures were %body fat and plasma total cholesterol. The secondary outcome measures were plasma TG, HDL-C, non-HDL-C, %HTR, atherosclerosis index, FFA, phospholipid, Apo A-1, Apo B, Apo B/Apo A-1, antioxidant enzyme activity and adipocytokine concentrations. Subjects were randomly assigned into three nutritional intervention groups, extract of *Glycine max *leaves (EGML; n = 28), *Garcinia cambogia *extract (GCE; n = 29) and placebo (n = 29). Assignment of subjects to nutritional intervention groups was conducted using randomly generated numbers. Both subjects and investigators were blinded to the nutritional intervention allocated. Subjects were instructed to maintain their routine food intake and physical activity throughout the course of the study.

### Protocol

At baseline and after the 10 week nutritional intervention subjects attended the Science Research Center laboratory at Kyungpook National University between 08:00 and 11:00 h after a 12-14 h overnight fast. Blood samples were drawn into heparin coated tubes, then centrifuged at 3000× g for 15 min at 4°C and stored at -70°C until plasma lipid, adipocytokine, antioxidant and toxicology analysis.

Waist girth was measured at the minimum circumference between the iliac crest and the rib cage. Hip girth was measured at the maximum width over the greater trochanters. Waist-to-hip ratio (WHR) was calculated as waist girth divided by hip girth. Systolic and diastolic blood pressure (BP) was measured using an automatic blood pressure monitor (Omron, Japan).

Body composition was determined with the X-Scan plus II body composition analyzer (Jawon Medical Company, Republic of Korea). The X-Scan plus II uses a 4-point tactile electrode system that measures the total and segmental impedance and phase angle of alternating electric current at 8-12 different frequencies. The X-Scan plus II software automatically calculates body composition based on tetrapolar bioelectrical impedance [23] with a proprietary formula: Total body water = A × Height^2^/Impedance + B × Weight + C × Age + D × Gender + E. Constants were derived from a validation study using the isotope dilution method. Before the body composition assessment, the subjects rested for 10 min, and then were asked to remove outer clothing (coats, sweaters), shoes and socks. Body weight was measured with a digital scale to the nearest 0.1 kg. Height was assessed with a stadiometer to the nearest 0.1 cm. Subjects were instructed to stand on the instrument's foot platforms and hold the palm-and-thumb electrodes, with their arms not touching their torso, while body composition was determined. Day-to-day reproducibility of bioelectrical impedance based body composition monitors to determine %body fat is reported to be 3.5-5% [24]. Body mass index (BMI) was calculated as weight(kg)/height(m^2^).

Food intake was recorded before and during the nutritional intervention trial using a 24 h dietary recall. Subjects were instructed to recall and describe the foods and beverages consumed over the previous 24 h. Subjects were asked to estimate portion sizes using common household bowls, cups and spoons. The 24 h dietary record for each subject was coded, and standard reference tables were used to convert estimated food portions to weight in grams. Nutritional analysis was performed using CAN-Pro 3.0 software (The Korean Nutrition Society), which provides a comprehensive database of the nutritional content of general foods and specialty Korean foods. The 24 h diet recall method has been reported to be a reliable and valid method to determine daily nutritional intake, which has been used in previous clinical trials [25,26].

### Nutritional intervention

Subjects consumed either four capsules containing EGML (2000 mg/day; Yuyu Health Care Co., Korea), eight capsules containing GCE (2000 mg/day; 60% hydroxyl citric acid; Newtree Inc., Korea) or four capsules containing placebo (2000 mg/day starch) in the morning and evening daily for 10 weeks. The total polyphenol content of EGML determined by HPLC (Shimadzu Corp., Japan) was 44.5 ± 2.1 mg gallic acid equivalents/g of EGML. The compliance of subjects to the nutritional intervention was regularly monitored every second day by telephone during the entire study period, all subjects reported consuming the supplement capsules as instructed. No serious adverse effects were reported by subjects consuming EGML, GCE or placebo supplements. There were no subject withdrawals from the study, therefore intention to treat analysis was not necessary.

### Plasma lipid and apolipoprotein analyses

Plasma total cholesterol (TC), triglyceride (TG), HDL-cholesterol (HDL-C), free fatty acid (FFA) and phospholipid (PL) concentrations were determined using commercially available kits based on enzymatic methods (Asan Pharm. Co., Korea). The ratio of HDL cholesterol to total cholesterol (HTR) was calculated as [(HDL-C/Total-Cholesterol × 100)]. Atherogenic index (AI) was calculated by [(Total-C - HDL-C)/HDL-C], Non-HDL-C was determined by [Total-C - HDL-C]. The apolipoprotin A-1 (Apo A-1) and apolipoprotein B (Apo B) were measured using commercial assay kits (ALerCHEK. Inc, USA).

### Plasma toxicology analysis

Plasma glutamic oxaloacetic transaminase (GOT) and glutamic pyruvic transaminase (GPT) were measured to assess toxicity of the EGML, GCE and placebo supplements. Plasma GOT and GPT activities were determined based on enzymatic methods using commercially available kits (Asan Pharm. Co., Korea).

### Oxidative stress and antioxidant analysis

To determine whether EGML and GCE supplementation provided protection against oxidative stress, the activity of the antioxidant enzymes catalase (CAT), glutathione peroxidase (GSH-Px) and superoxide dismutase (SOD) were measured in erythrocytes. To separate erythrocytes, EDTA treated blood samples were centrifuged at 3000× g for 15 min at 4°C. Erythrocytes were washed three times in 0.9% NaCl, lysed and mixed to produce a hemolysate. The hemoglobin concentration of the hemolysate was then estimated using a commercial assay kit (Asan Pharm. Co. Korea). SOD activity was measured according to the method of Marklund and Marklund [27]. SOD activity was expressed in unit/g hemoglobin. The CAT activity was measured using previously published methods [28]. A molar extinction coefficient of 0.041 (mM^-1^cm^-1^) was used to determine the CAT activity. CAT activity was defined as the reduction in μmol H_2_O_2_/min/g hemoglobin. GSH-Px activity was measured using the method described by Paglia and Valentine [29]. GSH-Px activity was expressed as oxidized μmol NADPH/min/g hemoglobin. As a marker of lipid peroxide production, the plasma thiobarbituric acid-reactive substances (TBARS) concentration was measured using the method of Tarladgis et al. [30].

### Plasma adipocytokine analysis

Adiponection, adipsin, leptin, resistin, TNF-α and IL-6 were measured in plasma samples, using multiplex detection kits (Bio-Rad, Hercules, USA). Data analyses were performed using the Bio-Plex Manager software version 4.1.1 (Bio-Rad, Hercules, USA).

### Statistical analysis

All data are presented as mean ± S.E. Statistical analysis was performed using SPSS software (version 11.5). Significant within group changes in body composition, plasma lipids, adipocytokine and antioxidant parameters between baseline and 10 weeks were assessed using paired Student's t-test. Significant differences between EGML, GCE and placebo supplemented groups at baseline and at 10 weeks were analyzed using one-way ANOVA. Post-hoc Duncan's multiple range tests were conducted when appropriate to further examine any significant between group differences at 10 weeks. Statistically significant differences were accepted at *p *< 0.05.

## Results

### Baseline characteristics

Baseline characteristics of subjects were not significantly different between EGML, GCE and placebo groups, although, the ratio of males to females was greater in the EGML group compared to the GCE and the placebo group. Fasting glucose and blood pressure of all overweight subjects were within the normal range (Table 1).

**Table 1:** Baseline clinical characteristics of subjects

Groups	Placebo	GCE	EGML
N (male: female)	29(15:14)	29(15:14)	28(16:12)
Age (y)	33.80 ± 2.97	34.07 ± 2.30	34.96 ± 2.42
Height (cm)	169.63 ± 2.84	167.17 ± 1.58	167.95 ± 1.78
Body weight (kg)	74.03 ± 3.03	71.09 ± 2.06	72.34 ± 1.92
BMI (kg/m^2^)	25.53 ± 0.43	25.29 ± 0.36	25.25 ± 0.37
Systolic BP (mmHg)	122.57 ± 4.49	125.69 ± 53	123.38 ± 4.02
Diasolic BP (mmHg)	76.85 ± 2.65	74.11 ± 3.66	75.45 ± 3.84
FBG (mg/dL)	90.04 ± 2.58	93.97 ± 3.44	88.73 ± 4.07
Waist (inch)	33.43 ± 0.69	32.43 ± 0.60	33.49 ± 0.61
Hip (inch)	39.49 ± 0.47	39.10 ± 0.42	39.54 ± 0.41

Values are mean ± S.E.; GCE, *Garcinia cambogia *extract; EGML, Extract of *Glycine max *leaves; FBG, fasting blood glucose

### Nutrient Intake

Baseline nutrient intake was not significantly different between groups (Table 2). Average energy intake of the groups was within current nutrient intake guidelines. Fat intake constituted around 15% of total energy intake and cholesterol intake was within the recommended guidelines. Subjects were instructed to maintain their habitual diet, however, 24 h diet recalls conducted during the trial revealed significantly higher cholesterol (p < 0.05) within each group pre- (Table 2) to post-supplementation (Table 3). Nevertheless there was no significant difference in energy, protein, carbohydrate, fat or cholesterol intake between the EGML, GCE and placebo groups at baseline or after 10 weeks supplementation (Table 2 and Table 3).

**Table 2:** Baseline nutrient intake

Groups	Placebo	GCE	EGML
Energy (Kcal/day)	2078.5 ± 96.9	1993.7 ± 102.9	2054.3 ± 118.2
Carbohydrate (g/day)	342.7 ± 51.2	338.9 ± 62.7	345.1 ± 55.8
Protein (g/day)	85.3 ± 6.7	79.7 ± 7.9	86.6 ± 4.4
Fat (g/day)	36.9 ± 4.7	35.6 ± 5.4	36.4 ± 6.9
Vit. B_1 _(mg/day)	1.3 ± 0.1	1.2 ± 0.1	1.1 ± 0.2
Vit. B_2 _(mg/day)	1.5 ± 0.1	1.2 ± 0.1	1.3 ± 0.2
Vit. C (mg/day)	61.6 ± 20.2	75.4 ± 11.8	69.6 ± 22.1
Ca (mg/day)	513.2 ± 52.9	508.1 ± 67.4	512.5 ± 57.2
Fe (mg/day)	13.8 ± 1.9	14.1 ± 1.9	12.9 ± 0.2
Cholesterol (mg/day)	223.8 ± 51.5	238.91 ± 17.0	226.66 ± 18.0
Fiber (g/day)	21.3 ± 3.6	26.5 ± 4.2	24.5 ± 0.9

Values are mean ± S.E.; GCE, *Garcinia cambogia *extract; EGML, Extract of *Glycine max *leaves

**Table 3:** Nutrient intake during the nutritional intervention

Groups	Placebo	GCE	EGML
Energy (Kcal/day)	2456.5 ± 116.9	2357.7 ± 92.9	2436.3 ± 101.9
Carbohydrate (g/day)	411.2 ± 60.1	388.9 ± 74.4	406.6 ± 72.1
Protein (g/day)	92.3 ± 6.8	86.7 ± 7.9	89.6 ± 4.5
Fat (g/day)	49.7 ± 8.0	50.6 ± 7.7	50.1 ± 8.3
Vit. B_1 _(mg/day)	1.6 ± 0.1	1.4 ± 0.1	1.3 ± 0.2
Vit. B_2 _(mg/day)	1.8 ± 0.1	1.2 ± 0.1	1.4 ± 0.2
Vit. C (mg/day)	65.6 ± 22.2	77.2 ± 10.8	70.2 ± 12.0
Ca (mg/day)	558.2 ± 53.8	523 ± 65.0	521 ± 52.3
Fe (mg/day)	14.5 ± 1.8	16.2 ± 1.5	13.5 ± 0.5
Cholesterol (mg/day)	557.8 ± 51.5	545.5 ± 11.0	550.0 ± 18.0
Fiber (g/day)	22.5 ± 4.5	27.6 ± 3.2	23.4 ± 0.6

Values are mean ± S.E.; GCE, *Garcinia cambogia *extract*; *EGML, Extract of *Glycine max *leaves

### Effect of EGML and GCE on body composition

There were no significant differences in body weight, body mass index (BMI) and waist-to-hip ratio (WHR) after 10 weeks supplementation with EGML or GCE compared to placebo (Table 4). There was a statistically significant change in %body fat and internal fat in the placebo group compared to both the EGML and GCE supplemented groups (p < 0.05; Table 4). However, the change in %body fat and internal fat was arguably of limited clinical significance. In addition, the change in %body fat may have been confounded by the lower %body fat in the placebo group.

**Table 4:** Effect of EGML or GCE on body composition in overweight subjects

Groups	Placebo		GCE				EGML		
	
	Before	After	Δ	Before	After	Δ	Before	After	Δ
Weight (kg)	74.03 ± 3.03	74.71 ± 3.06	0.68 ± 0.34	71.09 ± 2.06	71.73 ± 2.1	0.65 ± 0.43	72.34 ± 1.92	72.16 ± 1.97	-0.18 ± 0.30
BMI (kg/m^2^)	25.53 ± 0.43	25.77 ± 0.45	0.24 ± 0.11	25.29 ± 0.36	25.53 ± 0.40	0.24 ± 0.15	25.25 ± 0.37	25.49 ± 0.39	0.24 ± 0.11
WHR	0.85 ± 0.01	0.86 ± 0.01	0.01 ± 0.01	0.83 ± 0.01	0.84 ± 0.01	0.01 ± 0.01	0.85 ± 0.01	0.84 ± 0.01	-0.01 ± 0.01
BFP (%)	25.97 ± 1.68	27.36 ± 1.48	**1.39 ± 0.42^a^**	27.07 ± 0.76	27.74 ± 0.08	**0.67 ± 0.22^b^**	27.81 ± 0.93	27.65 ± 1.01	**-0.16 ± 0.24^b^**
Internal fat (kg)	2.45 ± 0.22	2.70 ± 0.23	**0.25 ± 0.07^a^**	2.42 ± 0.11	2.56 ± 0.12	**0.14 ± 0.04^b^**	2.54 ± 0.09	2.52 ± 0.10	**-0.02 ± 0.04^b^**

Values are mean ± S.E.; GCE, *Garcinia cambogia *extract; EGML, Extract of *Glycine max *leaves; WHR(Waist to Hip Ratio) = Waist/Hip, BFP, Body fat percentage; Δ: After-Before; **^ab^**Means in the same row not sharing a common superscript are significantly different among groups at p < 0.05 by one-way ANOVA;

### Effect of EGML and GCE on plasma lipid profiles

**Figure 1:**
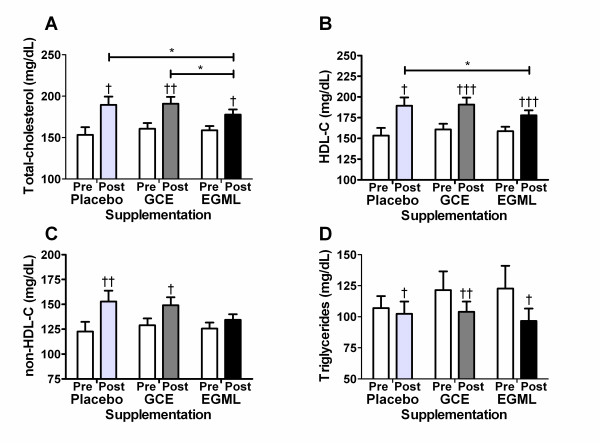
**Effect of EMGL or GCE supplementation on plasma (A) total-cholesterol, (B) HDL-C, (C) non-HDL-C and (D) triglycerides**. Values are mean ± S.E. *p < 0.05 indicates a between group difference. ^†^p < 0.05, ^††^p < 0.01, ^†††^p < 0.001 indicates a within group difference. GCE, *Garcinia cambogia *extract; EGML, Extract of *Glycine max *leaves.

At baseline there were no significant differences in plasma lipids between groups (Figure 1). Total cholesterol significantly increased in the EGML, GCE and placebo groups during the course of the trial (p < 0.05; Figure 1A). However, after 10 weeks supplementation total cholesterol was significantly lower in the EGML group (p < 0.05) compared to both the GCE and placebo group (Figure 1A). Plasma HDL-C concentration was increased significantly in all groups compared to baseline levels (p < 0.05), however after 10 weeks supplementation, HDL-C was significantly higher in the EGML compared to the placebo group (Figure 1B). Non-HDL-C concentration was increased significantly in only the placebo and GCE groups compared to baseline levels (p < 0.05; Figure 1C). However, non-HDL-C was not significantly different between groups after 10 weeks EGML or GCE supplementation (Figure 1C). Plasma TG was not significantly different in the EGML and GCE group compared to the placebo group after 10 weeks (Figure 1D). Plasma FFA and the Apo B/Apo A-1 ratio were significantly lower in the EGML group after 10 weeks supplementation compared to baseline, while %HTR level was significantly increased in the EGML group (p < 0.05) compared to baseline (Table 5). Unfortunately, neither EGML or GCE supplementation had any significant effect on FFA, PL, %HTR, Apo A-1, Apo B, Apo B/Apo A-1 ratio or atherosclerosis index after 10 weeks when compared to placebo (Table 5).

**Table 5:** Effect of EGML or GCE on plasma lipids and atherogenic biomarkers in overweight subjects

Groups	Placebo		GCE		EGML	
	
	Before	After	Before	After	Before	After
HTR (%)	21.36 ± 1.56	22.49 ± 1.94	21.08 ± 1.48	23.14 ± 1.38	20.43 ± 1.17	23.77* ± 1.16
AI	4.51 ± 0.58	4.31 ± 0.49	4.68 ± 0.33	3.82 ± 0.31	4.14 ± 0.35	3.43 ± 0.21
FFA (mmol/L)	0.54 ± 0.04	0.50 ± 0.03	0.49 ± 0.03	0.41 ± 0.03	0.49 ± 0.03	0.40* ± 0.02
PL (mmol/L)	1.58 ± 0.05	1.53 ± 0.05	1.61 ± 0.05	1.56 ± 0.05	1.64 ± 0.06	1.56 ± 0.04
Apo A-1 ( μg/dL)	1.82 ± 0.10	1.56 ± 0.04	1.72 ± 0.08	1.85 ± 0.12	1.66 ± 0.03	1.88 ± 0.15
Apo B ( μg/dL)	4.11 ± 0.33	4.10 ± 0.34	6.27 ± 0.63	5.55 ± 0.50	5.17 ± 0.40	4.70 ± 0.37
Apo B/Apo A-1	2.26 ± 0.63	2.62 ± 0.63	3.65 ± 0.42	3.00 ± 0.29	3.11 ± 0.23	2.50* ± 0.26

Values are mean ± S.E.; *p < 0.05, **< 0.01, ***p < 0.001 indicate significant difference before and after 10 weeks by Students' *t*-test; GCE, *Garcinia cambogia *extract*; *EGML, Extract of *Glycine max *leaves; HTR, [HDL-C/Total-C)×100]; AI, atherogenic Index, ([(Total-C)]-[HDL-C]/[HDL-C]); FFA, free fatty; PL, phospholipid; Apo A-1, Apolipoprotein A-1; Apo B, Apolipoprotein B.

### Effect of EGML and GCE on plasma toxicity and erythrocyte antioxidant enzyme activity

At baseline plasma GOT and GPT activities were within the normal healthy range across all groups (Table 6). After 10 weeks there was no evidence of increased plasma toxicity as a result of supplementation with EGML or GCE compared to placebo (Table 6).

**Table 6:** Effect of EGML or GCE on plasma toxicity biomarkers in overweight subjects

Groups	Placebo		GCE		EGML	
	
	Before	After	Before	After	Before	After
GOT (Karman/ml)	9.96 ± 1.45	12.73 ± 2.71	9.24 ± 1.07	8.61 ± 0.85	19.67 ± 8.86	11.23 ± 1.27
GPT (Karman/ml)	15.10 ± 3.57	15.38 ± 2.95	11.76 ± 1.50	10.15 ± 1.07	15.93 ± 2.91	15.49 ± 2.07

Values are mean ± S.E.; Data are compared between before and after 10 weeks by Students' *t*-test, and between groups before and after 10 weeks by one-way ANOVA; GCE, *Garcinia cambogia *extract*; *EGML, Extract of *Glycine max *leaves; GOT, glutamic oxaloacetic transaminase; GPT, glutamic pyruvic transaminase.

Antioxidant enzyme activity was not significantly increased by either EGML or GCE supplementation compared to placebo (Table 7). SOD and CAT activity remained unchanged in the EGML and GCE group compared to baseline. Although, there were significant differences in CAT activity between groups at baseline which may have confounded these results. GSH-Px activity was unchanged in the placebo group, but significantly increased as a result of 10 weeks EGML or GCE supplementation. However, neither EGML nor GCE supplementation resulted in higher GSH-Px activity compared to the placebo group. In addition, there was no evidence EGML or GCE supplementation altered oxidative stress, as plasma TBARS content remained unchanged compared to placebo (Table 7).

**Table 7:** Effect of EGML or GCE on erythrocyte and plasma antioxidant activity in overweight subjects

	Placebo		GCE		EGML	
	
	Before	After	Before	After	Before	After
**Erythrocytes**						
SOD (unit/mg Hb)	2.42 ± 0.07	2.19 ± 0.12	2.42 ± 0.07	2.40 ± 0.08	2.33 ± 0.06	2.30 ± 0.08
CAT (umol/min/mg Hb)	**1.38 ± 0.07^b^**	1.13 ± 0.10*	**1.22 ± 0.06^a^**	1.04 ± 0.08	**1.22 ± 0.05^ab^**	1.47 ± 0.30
GSH-Px (nmol/min/mg Hb)	2.33 ± 0.19	2.62 ± 0.27	2.16 ± 0.10	2.86 ± 0.28*	2.17 ± 0.13	2.93 ± 0.27*

**Plasma**						
TBARS (nmol/ml)	0.26 ± 0.02	0.27 ± 0.02	0.27 ± 0.02	0.30 ± 0.02	0.23 ± 0.01	0.25 ± 0.01

Values are mean ± S.E.; *p < 0.05 Data are compared before and after 10 weeks by Students' *t*-test; **^ab^**Means not sharing same letter are significantly different after 10 weeks between groups at p < 0.05 by one-way ANOVA; GCE, *Garcinia cambogia *extract*; *EGML, Extract of *Glycine max *leaves; SOD, superoxide dismutase; CAT, catalase; GSH-Px, glutathione peroxidase; TBARS, thiobarbituric acid-reactive substances.

### Effect of EGML and GCE on plasma adipocytokines

EGML and GCE supplementation had no effect on plasma adiponectin, adipsin, leptin, resistin, TNF-α or IL-6 concentrations compared to placebo (Table 8). Although, adipsin concentrations were significantly decreased over 10 weeks in the EGML and GCE groups compared to baseline (p < 0.05). While leptin was significantly increased in the placebo group, but was unchanged in the EGML and GCE groups compared to baseline (Table 8).

**Table 8:** Effect of EGML or GCE on plasma adipocytokines in overweight subjects

	Placebo		GCE		EGML	
	
	Before	After	Before	After	Before	After
Adiponectin ( μg/ml)	72.64 ± 4.66	72.05 ± 4.91	58.91 ± 4.45	71.25 ± 4.81	71.41 ± 5.25	69.97 ± 6.30
Adipsin ( μg/ml)	0.52 ± 0.04	0.50 ± 0.04	0.53 ± 0.03	0.43 ± 0.03*	0.56 ± 0.03	0.45 ± 0.03**
Leptin ( μg/ml)	12.64 ± 1.69	18.80 ± 2.31*	14.70 ± 1.77	14.35 ± 1.73	19.16 ± 4.81	17.89 ± 4.74
Resistin ( μg/ml)	4.42 ± 0.31	5.65 ± 0.63	4.82 ± 0.34	4.79 ± 0.48	4.31 ± 0.25	4.88 ± 0.38
TNF-α (pg/ml)	6.92 ± 0.44	7.87 ± 0.52	7.68 ± 0.55	7.26 ± 0.57	7.93 ± 0.70	7.30 ± 0.52
IL-6 (pg/ml)	9.50 ± 0.90	9.57 ± 0.81	9.07 ± 0.47	9.29 ± 0.67	8.91 ± 0.57	9.93 ± 0.55

Values are mean ± S.E.; *p < 0.05, **p < 0.01, ***p < 0.001: Data are compared before and after 10 weeks by Students' *t*-test. ^ab^Means not sharing same letter are significantly different after 10 weeks between groups at p < 0.05 by one-way ANOVA. GCE, *Garcinia cambogia *extract*; *EGML, Extract of *Glycine max *leaves.

## Discussion

Human nutritional intervention studies are important to evaluate the potential health benefits of natural supplements suggested from evidence based on animal studies. This study was designed to determine the effectiveness of a *Glycine max *(EGML) based supplement to promote weight-loss and lower plasma cholesterol compared to *Garcinia cambogia *(GCE) or placebo supplementation.

### EGML or GCE supplementation does not promote weight-loss

The present study indicated EGML or GCE supplementation was not effective in promoting weight-loss in overweight individuals. BMI and waist-to-hip ratio are important risk factors associated with cardiovascular disease risk, but neither EGML supplementation nor GCE supplementation had any significant effect on either BMI or waist to hip ratio in the overweight individuals. Waist to hip ratio is a crude indicator of abdominal adiposity which represents visceral fat accumulation, although used in the diagnosis of metabolic syndrome, waist to hip ratio may not be sufficiently sensitive to detect small changes caused by a nutritional intervention.

Body composition parameters are more sensitive than BMI for detecting small changes in body fat, as individuals with the same BMI can have a wide variability in body fat. Indeed we found the body composition analysis revealed the %body fat change was statistically lower in the EGML and GCE supplemented group compared to placebo after 10 weeks. However, it is important to emphasize that a 1-1.5% body fat change is arguably of no clinical relevance to disease risk. In addition, body composition methods based on bioelectrical impedance typically have day-to-day reproducibility between 3.5-5% [31], which should be taken into consideration when evaluating the effectiveness of natural food supplements to promote weight-loss.

Previous studies showing soy supplementation can promote weight-loss were mainly conducted in conjunction with calorific restriction, and involved soy protein with isoflavonoids [11]. In contrast in the present study we used a soy leaves supplement which contained abundant flavonoids but minimal protein, in addition subjects consumed their habitual diet. On the basis of the present findings neither EGML nor GCE supplementation alone can promote weight-loss in overweight individuals. These findings are in agreement with the most recent meta-analyses of randomized control trials of GCE or soy flavonoids which report minimal or no effects of GCE or soy flavonoids on weight-loss in humans [11,14]. Clearly, a natural food based supplement which is able to promote weight-loss would be of significant clinical benefit [5]. It is unknown whether longer EGML or GCE supplementation over 6-12 months may lead to a clinically significant reduction in body fat accumulation.

### No effect of EGML or GCE supplementation on energy intake

Natural food supplements such as EGML are purported to increase satiety, therefore may help reduce calorie intake, but the present study and previous human studies on soy supplementation show no effect on satiety or calorie intake [32,33]. We actually observed an increase in both energy and cholesterol consumption within all groups during the study. One explanation may have been dietary intake was under-reported at baseline. Lack of familiarity and poor memory may both contribute to under-reporting when using a 24 h dietary recall [34]. A recent European study suggested conducting multiple dietary recalls at baseline can help to ensure dietary recall is reliable and reflects actual dietary intake [34]. Remarkably the increase in cholesterol intake evident in the EGML, GCE and placebo groups over 10 weeks was also reflected in plasma total cholesterol levels within all groups. In agreement with past studies the present study provided no evidence that EGML or GCE supplementation can modify calorie intake in overweight individuals consuming their habitual diet [11].

### EGML and GCE supplementation can modify plasma lipid profiles in overweight individuals

Lowering total cholesterol, triglyceride, LDL-C or non-HDL-C concentration is reported to decrease the risk of developing atherosclerosis [4]. Obesity is strongly associated with elevated plasma cholesterol and lipoproteins. Hence natural food supplements which can significantly improve plasma lipid profiles may be useful for cholesterol management and prevention of atherosclerosis [10]. In the present study, EGML supplementation resulted in ~6% lower total plasma cholesterol compared to both the GCE and placebo supplemented groups after 10 weeks, although after 10 weeks total plasma cholesterol was actually higher than baseline in all groups. One explanation for the increase in total plasma cholesterol was the increase in cholesterol intake evident from the 24 h dietary recall analysis. The minimal effect of EGML on plasma cholesterol accumulation compared to placebo is arguably of limited clinical significance given that total plasma cholesterol was considerably higher after 10 weeks.

Plasma non-HDL-C provides a measure of multiple lipoproteins including LDL. The lipoproteins present in non-HDL-C are responsible for transporting cholesterol and lipids in the blood. In the 1990's LDL-C was the primary target of cholesterol reduction programs [4]. A review of clinical studies reported for every 1% reduction in LDL-C levels, relative risk for coronary heart disease (CHD) events was reduced by approximately 1% [4]. At baseline the overweight subjects included in this study had a plasma non-HDL-C level (equivalent to LDL-C) between 122-129 mg/dl. The National Cholesterol Education Program (NCEP) ATP III guidelines recommend cholesterol lowering treatment in high-risk individuals with LDL-C above 130 mg/dl [4]. During the study, subjects consumed their habitual diet, we observed plasma non-HDL-C actually increased in the placebo and GCE groups to ~150 mg/dl after 10 weeks. Inter-individual variability in non-HDL-C was evident in all groups, which may be partly due to diet but also genetic variation. Some overweight individuals with high non-HDL-C are resistant to statin treatment [4], therefore developing nutritional interventions using natural food supplements may provide a way to further lower non-HDL-C and hence risk of cardiovascular disease.

Elevated plasma triglycerides in overweight patients, are not as strongly associated with increased cardiovascular risk compared to plasma cholesterol, but nevertheless are clinically significant as elevated plasma triglyceride levels are an indicator of hepatosis and dyslipidemia [35]. In the present study neither EGML nor GCE supplementation significantly reduced plasma triglyceride levels. Although, plasma triglycerides were ~15-20% lower in the EGML and GCE groups after 10 weeks compared to baseline, again wide inter-individual variability in plasma triglyceride levels may explain the lack of a significant detectable reduction compared to placebo.

Risk of cardiovascular disease is inversely associated with plasma HDL-C concentrations and hence increased HDL may help protect against atherosclerosis [36]. At baseline all overweight subjects had a low HDL < 40 mg/dl based on NCEP ATP III guidelines [4]. However, after 10 weeks the EGML group had significantly higher plasma HDL-C levels compared to the placebo group, and hence decreased risk of cardiovascular disease. Prospective epidemiological studies have consistently indicated that high levels of plasma HDL-C can protect against the development of atherosclerosis and cardiovascular disease [37]. EGML appears to be effective for increasing plasma HDL-C levels in overweight individuals. In contrast, GCE supplementation appeared to be ineffective for raising HDL-C compared to placebo in overweight individuals.

### No evidence of toxicity after EGML or GCE supplementation in overweight individuals

Despite the widespread use of nutritional supplements their safety and toxicity are rarely tested in humans. Plasma GOT and GPT activity provides an indicator of hepatoxicity [38]. At baseline plasma GOT and GPT activity was within the normal range and not significantly altered by 10 weeks EGML supplementation in overweight individuals. Furthermore, no subjects in the study reported adverse side-effects due to EGML supplementation. Nevertheless some studies have reported that natural food supplements containing high flavonoid doses far in excess of dietary intake may have unwanted biological effects on absorption of other nutrients and trace elements [39]. Potential interactions between drugs and nutritional supplements remain largely unknown. Therefore, it is advisable that natural food supplement consumption in overweight individuals is supervised by a medical physician.

### EGML or GCE supplementation do not modify antioxidant activity or adipocytokine levels in overweight individuals

Soy based supplements are reported to have antioxidant effects in animals [40] and humans [41]. Furthermore, a clinical study in pre- and post-menopausal Korean women reported soy intake was inversely correlated with oxidative stress (TBARS) [42]. Oxidative stress may play a role in the pathogenesis of atherosclerosis, as endothelial damage by lipid particles can lead to production of reactive oxygen species, macrophage infiltration and the development of an atherosclerotic plaque [43]. In the present study there was a trend for EGML to increase CAT and GSH-Px activity in erythrocyte after 10 weeks supplementation, but these results appeared to be confounded by inter-individual variability in antioxidant enzyme activity. EGML and GCE supplementation did not significantly effect plasma TBARS level either which is a marker of lipid peroxidation. An important caveat was that the overweight individuals in this study did not show any evidence of excess oxidative stress at baseline, compared to other human obesity studies [44]. The present findings do not preclude the possibility that EGML or GCE may possess antioxidant activity in overweight individuals who have pre-existing high levels of oxidative stress.

Plasma adipocytokine levels are reported to be associated with BMI and adiposity [45]. In concordance with the lack of clinically significant changes in body composition, we observed no change in adipocytokine levels in the EGML or GCE group compared to placebo.

### Study limitations

Firstly, the influence of sex differences on the effect of EGML or GCE on primary and secondary outcomes was not determined. Hormonal changes during the menstrual cycle are a potential confounding factor in this study. Plasma cholesterol, TG and LDL-C are reported to vary between ~5-8% during the follicular stage [46]. To minimize potential sex differences subjects were randomized into treatment and placebo groups, despite randomization there was still an imbalance in males and females in each group.

Secondly, it was beyond the scope of the present study to determine whether differences in soy metabolism between subjects in the EGML group may have influenced the outcome. Some reviews suggest only 30-50% of humans have the bacteria capable of producing equol which is the primary metabolite of the soy isoflavone daidzein [47]. Therefore, inter-individual variability in equol activity may explain the lack of significant effects of EGML supplementation on primary outcomes compared to placebo [47].

Soy intake of Asian populations is reported to be higher than average soy intake in large European or American cohort studies [48]. Also *Garginia cambogia *is a food component used widely in South-East Asia. Therefore, soy and GCE based supplements may have smaller effects if any in randomized control trials conducted in Asia. In agreement with previous studies on soy and GCE supplements we used doses of 2 g/day, it was beyond the scope of the present study to determine whether higher doses were more effective for promoting weight-loss and improving plasma lipid profiles. Although, a previous report suggest higher doses may be futile, as oral administration of higher doses of *Garcinia cambogia *extract to normal subjects leads to increased urinary excretion of (-)-hydroxycitric acid, attributed to limitations in (-)-hydroxycitric acid absorption efficiency [49].

It is important in longitudinal nutritional intervention studies targeting body fat reduction that the body composition method has acceptable reliability and validity. Small changes in %body fat need to be interpreted with caution as daily or weekly intra-individual variability in bioelectrical impedance monitors is reported to range from 3.5-5% [31]. Furthermore, regression to the mean may have occurred despite randomization of treatments, and contributed to within-group and between-group variation in baseline clinical measures and should be considered when interpreting the findings [50,51]

## Conclusions

Ten weeks EGML or GCE supplementation failed to promote any clinically significant weight-loss and had a minimal effect on %body fat in overweight individuals consuming their habitual diet. Although, EGML or GCE supplementation improved plasma HDL-C in overweight individuals, neither EGML nor GCE had any clinically significant effects on other plasma lipids, antioxidant or adipocytokine levels compared to placebo. More randomized human trials of natural products suggested to improve health are essential to establish actual efficacy, which will help to facilitate evidenced based dietary supplementation.

## Abbreviations

EGML: extract of *Glycine max *leaves; GCE: *Garginia cambogia *extract

## Competing interests

The authors declare that they have no competing interests.

## Authors' contributions

MSC, TSJ, WSL, KHP conceived and designed the study. KHP, WSL and TSJ prepared the nutrition supplement and chemical analysis. JEK and SMJ conducted the nutritional intervention and blood analysis. JEK performed the statistical analysis. JEK, RAM and MSC made significant intellectual contribution, interpreted the results and wrote the manuscript. All authors read and approved the final manuscript.
